# Translation of surface electromyography into a clinically applicable objective bulbar assessment tool to improve measurement-based care in amyotrophic laterals sclerosis

**DOI:** 10.3389/fnins.2026.1784520

**Published:** 2026-04-10

**Authors:** Panying Rong, Lindsey Heidrick, Gary Pattee

**Affiliations:** 1 Department of Speech-Language-Hearing: Sciences and Disorders, University of Kansas, Lawrence, KS, United States; 2 Department of Hearing and Speech, University of Kansas Medical Center, Kansas City, KS, United States; 3 Neurology Associates P.C., Lincoln, NE, United States

**Keywords:** amyotrophic lateral sclerosis, automated analysis, biomarker, bulbar involvement, machine learning, neurodegenerative disease, objective measurement, surface electromyography

## Abstract

**Objective:**

This study aims to translate surface electromyography (sEMG) into a clinically applicable, objective tool for assessing bulbar involvement in amyotrophic lateral sclerosis (ALS).

**Methods:**

A clinically grounded sEMG framework was developed, integrating a standardized, repeatable protocol with a novel analytic pipeline, to automatically extract 60 features from six craniofacial muscle groups during a set of motorically demanding but cognitively and linguistically less challenging oral diadochokinetic (DDK) tasks. Using this framework, 104 oral DDK recordings were acquired from 16 individuals with ALS—nine with overt bulbar symptoms (ALS+B) and seven without (ALS-B)—and 10 healthy controls (HCs). The sEMG features were clustered into 10 interpretable composite measures and validated by evaluating their (1) internal consistency using Cronbach’s α ; (2) associations with standardized functional outcomes and a biomechanical metric—stiffness—via mediation analysis; (3) discriminatory efficacy in distinguishing ALS+B and ALS-B from HC, as well as from each other, using machine learning classifications; and (4) robustness to common nonmotor confounders, including age, sex, and cognitive-linguistic impairments, through a comparison of discriminatory performance before and after adjustment for these factors.

**Results:**

All composite measures exhibited (1) high internal consistency (Cronbach’s α=0.89±0.071 ), (2) significant (or marginally significant) direct or stiffness-mediated indirect associations with the functional outcomes, and (3) consistently high discriminatory accuracy (0.82–0.85), both before and after adjustment for confounders.

**Conclusion:**

The sEMG framework demonstrates strong potential as a reliable, valid, and robust objective tool to detect subclinical neuromuscular changes throughout the prodromal and symptomatic phases of bulbar involvement in ALS, while remaining resistant against disease-related cognitive-linguistic impairments and disease-unrelated confounders. This tool may augment standard clinical evaluations, enabling earlier detection of bulbar involvement and measurement-based care in ALS.

## 1 Introduction

Amyotrophic lateral sclerosis (ALS) is a debilitating neurodegenerative disease associated with the dynamic propagation of pathological proteins across neural networks, leading to progressive degeneration of both upper and lower motor neurons (UMNs and LMNs) in the brain and spinal cord (Armon, 2008). The resulting pathophysiological changes usually start focally and progressively extend throughout the skeletal musculature over time. Among the diverse functional impairments associated with ALS, progressive loss of speech communication due to bulbar involvement—a hallmark feature affecting the craniofacial musculature—has been consistently identified by patients as the most devastating consequence, profoundly diminishing quality of life (Hecht et al., 2002). Effective management of communication disorders in ALS requires timely, individualized interventions to help patients maintain functional communication and social engagement throughout the disease course, thereby maximizing their quality of life (Yorkston et al., 2002). Grounded in the broader framework of personalized medicine, this management approach highlights two priorities in the assessment of bulbar involvement: (1) early diagnosis and referral to ensure timely access to optimal care, and (2) regular re-evaluations to enable proactive adaptation of interventions to patients’ evolving needs throughout the disease course (Fried-Oken et al., 2015).

The pathophysiology of bulbar involvement in ALS is associated with complex neuromuscular changes that initially emerge at subclinical levels during the prodromal phase and progressively advance to overt clinical symptoms and functional declines during the symptomatic phase (Eisen et al., 2014). Early detection and dynamic monitoring of these changes across both phases is essential for the effective management of bulbar involvement. However, current clinical standards for bulbar assessment—integrating neurological exams, patient- and clinician-reported outcomes, and functional speech and swallowing assessments—rely on subjective, non-standardized, or lengthy procedures and lack sensitivity to subclinical changes, especially during the prodromal phase (Pattee et al., 2019; Yunusova et al., 2019). To address these limitations, there is an urgent need for reliable and valid objective methods capable of detecting subtle yet meaningful subclinical changes in bulbar neuromuscular mechanisms to complement standard clinical evaluations.

The goal of this study is to establish a clinically applicable surface electromyography (sEMG) framework for objective assessment and characterization of subclinical neuromuscular changes throughout the prodromal and symptomatic phases of bulbar involvement in ALS. sEMG is a clinically accessible electrophysiological technique with strong potential as a noninvasive, objective, and quantifiable tool for assessing neuromuscular impairments and their associated functional alterations (Campanini et al., 2020). In contrast to intramuscular electromyography—an electrodiagnostic tool that relies on expert-guided invasive procedures to detect LMN pathology by evaluating a highly selective subset of motor units—sEMG records action potentials from a larger pool of motor units under the targeted skin area, providing a more comprehensive view of the neuromuscular mechanisms underlying force generation, including recruitment, rate coding, and synchronization. All these mechanisms are vulnerable to both UMN and LMN degeneration; therefore, sEMG is ideally suited to assess the combined effects of UMN and LMN pathologies on neuromuscular mechanisms. Despite its well-recognized theoretical potential, sEMG has remained largely underutilized in clinical practice to date. The major barriers include (1) a lack of standardized protocols, including guidelines for task selection, electrode placement, and instrumentation; (2) limited clinician expertise in interpreting sEMG data, which can be alleviated through the development of user-friendly analytic algorithms that automatically extract clinically meaningful information; and (3) a paucity of robust evidence demonstrating the added value of sEMG over standard clinical evaluations, which has constrained the interests of clinical stakeholders in investing in the technology (Campanini et al., 2020).

This study aims to overcome existing barriers and translate sEMG into a clinically applicable, objective, and quantifiable tool for assessing bulbar involvement in ALS. To achieve this goal, we developed a craniofacial sEMG framework that integrates a standardized, repeatable protocol with a user-friendly analytic pipeline for automated extraction of objective, clinically meaningful information from craniofacial sEMG signals. The protocol was designed around the strategic selection of (1) craniofacial muscle groups with well-defined anatomical landmarks to ensure reproducible electrode placement and high-quality data acquisition, and (2) oral diadochokinetic (DDK) tasks that preserve the strengths of previously investigated speech tasks—including their sensitivity to bulbar involvement in ALS (Rong et al., 2025; Rong and Jawdat, 2021; Rong and Pattee, 2021; Rong and Pattee, 2022)—while reducing cognitive-linguistic demands to enhance the robustness of task performance to cognitive-linguistic deficits that frequently cooccur with bulbar involvement as a consequence of frontotemporal lobar degeneration in ALS (Bak and Hodges, 2004; Goldstein and Abrahams, 2013). The analytic pipeline incorporated multiple scientifically and clinically grounded algorithms that have been individually validated in our prior studies (Rong et al., 2025; Rong and Pattee, 2022), with the goal of providing a comprehensive and fine-grained assessment of subclinical changes in craniofacial neuromuscular performance in ALS.

Using the proposed sEMG framework, we first evaluated its reliability and validity and then demonstrated its added value over standard clinical evaluations in detecting subtle subclinical neuromuscular changes, including early changes emerging during the prodromal phase of bulbar involvement. It was hypothesized that the sEMG framework would offer a reliable and valid tool to complement standard clinical evaluations by enabling earlier detection of prodromal bulbar involvement and providing novel objective measures capable of capturing clinically indiscernible yet meaningful neuromuscular changes.

## 2 Materials and methods

This study was conducted as part of a larger, ongoing project, with all study protocols approved by the Institutional Review Board of the university medical center. Written informed consent was obtained from all participants. All study procedures were noninvasive with minimal risk. No adverse events were reported.

### 2.1 Participants

The participants for this study included 16 individuals with ALS—nine with overt clinical bulbar symptoms (ALS+B) and seven without (ALS-B)—and 10 neurologically healthy controls (HCs). Participants with ALS were recruited from the multidisciplinary ALS clinic at the university medical center, meeting the following inclusion criteria: (1) being diagnosed with definite or probable ALS in accordance with the revised El Escorial Criteria (Brooks et al., 2000); (2) speaking Americal English as the first and primary language; (3) passing a hearing screening at 1,000, 2,000, and 4,000 Hz at 30 dB in the better ear; (4) preserving adequate cognitive capacity to follow instructions and comply with study procedures, with a score ≥ 20 on the Montreal Cognitive Assessment (MoCA) (Nasreddine et al., 2005); and (5) reporting no depression or other psychiatric disorders.

Prior to study participation, participants with ALS underwent standard clinical evaluations of bulbar motor function that combined clinician-administered procedures, including an oral mechanism examination, dysarthria screening, perceptual speech analysis, as well as swallow-related screening (e.g., 3 oz. water test) and bedside and/or instrumental evaluations, with patient-reported outcomes using standardized questionnaires (e.g., Eating Assessment Tool; Belafsky et al. (2008)). Based on these clinician-based evaluations and patient-reported outcomes, participants were classified as ALS-B if they exhibited no overt speech or swallowing symptoms, and as ALS+B otherwise. Given prior evidence showing over 90% likelihood of subclinical bulbar involvement at the time of ALS diagnosis in a heterogeneous cohort (77% with non-bulbar onsets) (Rong et al., 2015), the ALS-B subgroup in this study—on average 453 days post-diagnosis—is expected to experience subclinical bulbar involvement, thereby representing the prodromal phase of bulbar involvement. In contrast, the ALS+B subgroup represents the symptomatic phase.

During the study session, participants with ALS completed the ALS Functional Rating Scale-Revised (ALSFRS-R)—a 12-item questionnaire that assesses the severity of disability across gross and fine motor, bulbar, and respiratory domains (Cedarbaum et al., 1999). The sum of subscores on the bulbar items (i.e., speech, swallowing, salivation) was calculated to index the severity of bulbar functional disability. In addition, all participants completed a standardized functional speech assessment—the Sentence Intelligibility Test (Yorkston et al., 2007)—in which they read 11 randomly generated sentences varying in length from 5 to 15 words. Two adult listeners who are native speaker of American English with normal speech, language, hearing, and cognitive functions transcribed and timed the recorded speech samples. Based on the mean responses between listeners, two standardized functional speech metrics—speech intelligibility and speaking rate—were calculated as the percentage of correctly transcribed words and the number of words per minute, respectively. The clinical, functional, and demographic characteristics of the participants are summarized in Table 1.

**Table 1:** Participants characteristics.

Participant characteristics		ALS (*N* = 16)	HC (*N* = 10)	Group comparison: ALS vs. HC
Demographic characteristics				
Women (*N*%)		37.50%	70.00%	$\chi^{2}=1.46$ , p=0.23
Age, years (*M*; SD)		58.75; 14.58	66.80; 13.02	t=−1.46 , p=0.16
Overall clinical characteristics				
Time since diagnosis, days (*M*; SD)		374.06; 404.15	n.a.	n.a.
Disease onset (*N*_bulbar_; *N*_spinal_)		5;11	n.a.	n.a.
ALSFRS-R total score (M; SD)		37.50; 6.37	n.a.	n.a.
MoCA score (*M*; SD)		25.56; 3.31	26.75; 1.28	t=−1.26 , p=0.22
Bulbar characteristics				
Clinical manifestation (*N*_ALS-B_; *N*_ALS+B_)		7; 9	n.a.	n.a.
ALSFRS-R	Bulbar subscore (*M*; SD)	9.69; 2.80	n.a.	n.a.
	Speech subscore (*M*; SD)	3.13; 1.03	n.a.	n.a.
	Swallowing subscore (*M*; SD)	3.38; 0.96	n.a.	n.a.
	Salivation subscore (*M*; SD)	3.19; 1.17	n.a.	n.a.
Speech intelligibility, % (*M*; SD)		83.66; 30.15	99.45; 0.56	t=−2.10 , p=0.054
Speaking rate, words per minute (*M*; SD)		132.86; 42.37	183.65; 23.02	t=−3.95 , p<0.001 *

ALS, amyotrophic lateral sclerosis; HC, healthy control; MoCA, Montreal Cognitive Assessment; ALSFRS-R, Amyotrophic Lateral Sclerosis Functional Rating Scale-Revised; ALS-B, amyotrophic lateral sclerosis without overt clinical bulbar symptoms; ALS+B, amyotrophic lateral sclerosis with overt clinical bulbar symptoms; n.a., not applicable.

### 2.2 Protocol

#### 2.2.1 Tasks

Participants performed four oral diadochokinetic (DDK) tasks, each consisting of the rapid repetitions of a single syllable ([pɑ], [tɑ], or [kɑ]) or a multisyllabic unit ([pɑtɑkɑ]) at their maximum rate within one breath. These tasks were selected for their (1) high motoric demand on craniofacial muscles, requiring rapid, repetitive contractions that enhance their susceptibility to subtle motor deficits (Rong, 2020; Rong and Rasmussen, 2024; Rong et al., 2018), and (2) low cognitive-linguistic demands due to their simple phonological structures and lack of semantic and syntactic elements. Moreover, oral DDK tasks are feasible for individuals across a wide range of bulbar severity, including those with marked reduced intelligibility that limits their ability to perform complex speech tasks. Task compliance was monitored by the experimenter throughout the session, and any recordings with violations of instructions were excluded and subsequently reacquired.

#### 2.2.2 Target muscle groups

Three bilateral muscle groups—masseter, temporalis, and submental complex—were selected. Prior studies have identified measurable jaw motor deficits across both prodromal and symptomatic phases of bulbar involvement in ALS (Rong, 2021; Rong and Jawdat, 2021; Rong and Pattee, 2021), making the muscles of the jaw appropriate targets for bulbar assessment. Moreover, the selected muscles are accessible via surface electrodes; when recorded using a standardized protocol, surface-detected signals from these muscles have been shown to be reliable, reproducible, and comparable to those obtained with intramuscular electrodes during cyclic jaw movements (Koole et al., 1991; Musto et al., 2017; Suvinen et al., 2009).

#### 2.2.3 Experimental setup and instrumentation

A portable wireless sEMG system (BIOPAC) was used to record myoelectric activities of the selected muscles. In accordance with best-practice guidelines for sEMG implementation (Merletti and Cerone, 2020; Merletti and Muceli, 2019; Stepp, 2012), the target skin areas were shaved and cleansed with an alcohol swab to increase skin conductance. Small self-adhesive, pre-gelled Ag/AgCl electrodes (11 mm diameter) were placed in a bipolar configuration with an interelectrode distance of 20 mm (or one-quarter the length of the muscle fiber, when applicable) on the belly of each muscle, parallel to the fiber orientation. To maximize reproducibility, craniofacial anatomical landmarks were used to guide electrode placement: (1) temporalis, aligned vertically along the coronal suture (Musto et al., 2017); (2) masseter, oriented parallel to the cantho-gonial line, with the upper pole of the electrode under the tragus-labial commissural line (Musto et al., 2017); and (3) submental complex, aligned parallel to the anterior belly of the digastric, approximately 40% of the distance between the mentis and hyoid bone (Oommen and Kim, 2018). Ground electrodes were attached to the shoulder. Electrode placement was verified with (1) teeth clenching to elicit temporal and masseter activities, (2) jaw lowering against resistance and saliva swallowing to elicit submental complex activity, and (3) jaw oscillations to elicit dynamic contractions of all targeted muscles (O'Dwyer et al., 1981). Analog sEMG signals were pre-amplified ( × 2,000), band-pass filtered (5–500 Hz), and digitized at 2,000 Hz for acquisition. Audio recordings were simultaneously obtained at 22,050 Hz using a head-mounted microphone (dfine 4,188; DPA) placed approximately 5 cm from the left lip corner to assist with sEMG data handling and interpretation.

#### 2.2.4 Signal quality control: normalization, crosstalk attenuation, and filtering

To minimize individual variability arising from disease-unrelated physical (e.g., electrode contact), anatomical (e.g., skinfold thickness), and physiological (e.g., body temperature) factors, the raw sEMG signals were normalized to reference values during a maximum voluntary contraction (MVC) test. In this test, participants (1) clenched the teeth as forcefully as possible for 5 s to elicit MVC of the temporalis and masseter muscles, and (2) lowered the jaw against resistance as forcefully as possible for 5 s to elicit MVC of the submental complex, while maintaining a relaxed facial expression and minimal head and neck movements (O'Dwyer et al., 1981; Stepp, 2012). The test was repeated three times, with 1-min breaks between trials. The root-mean-square (RMS) value of the most stable 3-s interval from each trial was calculated using 500-millisecond moving windows. The mean RMS across trials was used as reference values for normalizing sEMG signals.

To minimize interferences and artifacts, a blind source separation algorithm (Kilner et al., 2002) was applied to attenuate crosstalk in the sEMG signals. Moreover, all sEMG signals were notch-filtered at 60 Hz and high-pass filtered at 20 Hz to attenuate power line interferences, low-frequency motion artifacts, and baseline wander (De Luca et al., 2010).

### 2.3 Analytic pipeline

The analytic pipeline automatically extracted 60 features from the processed sEMG signals to quantify six constructs of craniofacial neuromuscular performance, including the frequency, amplitude, complexity, and visibility of individual muscle activity, as well as the functional connectivity and integration of the entire muscle network. As outline in Table 2, this pipeline includes five analyses. All analyses were implemented in MATLAB (R2024b, MathWorks), with technical details provided below.

**Table 2:** Surface electromyography features.

Construct	Analysis	Feature (# of features)
Amplitude	Linear time-domain analysis	Mean absolute value (6)
		Waveform length (6)
Frequency	Linear frequency-domain analysis	Mean power spectral frequency (6)
	Linear time-domain analysis	Zero crossing rate (6)
		Slope sign change (6)
Complexity	Recurrence quantification analysis	Recurrence rate (6)
		Determinism (6)
	Nonlinear time-frequency domain analysis	Shannon entropy (6)
Visibility	Graph network analysis	Density (6)
Functional connectivity	Graph network analysis	Mean nodal strength (3)
Functional integration	Graph network analysis	Global efficiency (3)

#### 2.3.1 Linear time-domain analysis

Linear time-domain analysis was applied to 100-ms segments centered on the bursts in each channel, which encode the most stable and useful neuromuscular information. The following features were extracted from individual segments and then averaged across all segments for each sEMG signal using Equation (1)-(4):

(i) Mean absolute value (MAV): mean absolute value of the amplitudes of all data points within a segment:

$$\mathrm{MAV}=\frac{1}{N}\sum_{i=1}^{N}|{\dot{x}}_{i}|$$ (1)

where *N* is the number of data points in the segment, and $x_{i}$ is the amplitude of the *i*th data point in the segment.

(ii) Waveform length (WL): cumulative length of the waveform over the segment:

$$\mathrm{WL}=\sum_{i=1}^{N-1}|x_{i+1}-x_{i}|$$ (2)

(iii) Zero crossing rate (ZR): number of times the waveform crosses zero:

$$\mathrm{ZR}=\sum_{i=1}^{N-1}[f(-x_{i}\times x_{i+1})\times |x_{i}-x_{i+1}|\geq 4\;\mathrm{mV}]$$ (3)

where $f(x)=\{\begin{array}{c} 1,x>0\\ 0,x\leq 0 \end{array}$ . To eliminate crossings due to noise fluctuations, the threshold was set as 4mV (i.e., 4μV peak-to-peak intrinsic system noise ×2,000 gain /2 ) instead of zero.

(iv) Slope of sign change (SlpSignChange): number of times the slope of the waveform changes sign:

$$Sl\text{pSignChange}=\sum_{i=2}^{N-1}f[(x_{i}-x_{i-1})\times (x_{i}-x_{i+1})\geq 4\;\mathrm{mV}]$$ (4)

Similar as above, a 4mV threshold was employed to eliminate smaller noise-induced sign changes.

#### 2.3.2 Linear frequency-domain analysis

Linear frequency-domain analysis computed the power spectral density for each sEMG signal, using Welch’s averaged, modified periodogram method, implemented with 1,024-point Fast Fourier Transform (FFT) across 100-msec, 50% overlap sliding Hamming windows. Mean power spectral frequency (MNF) was calculated using Equation (5) as follows:


$$\mathrm{MNF}=\frac{\sum_{i=1}^{N}f_{i}P_{i}}{\sum_{i=1}^{N}P_{i}}\;\;$$ (5)

where $f_{i}$ and $P_{i}$ are the frequency and power at the *i*th frequency bin of the power spectrum and N is the total number of frequency bins.

#### 2.3.3 Nonlinear time-frequency domain analysis

First, each sEMG signal was decomposed into eight sub-band signals through iterated low- and high-pass filtering, using a 3-level wavelet packet decomposition with the 5th order Symlets wavelet. Next, the Shannon entropy of each sub-band signal was computed using Equation (6) as follows:


$$\text{ShanEn}=-\sum_{i}{s_{i}}^{2}\log({s_{i}}^{2})$$ (6)

where $s_{i}$ is the *i*th coefficient of the sub-band signal, representing the approximation (i.e., low-pass) or detail (i.e., high-pass) coefficients at each decomposition level. Lastly, within each channel, the sum of Shannon entropy across all sub-band signals was calculated, with higher values reflecting greater irregularity and, consequently increased complexity of the sEMG signal.

#### 2.3.4 Recurrence quantification analysis

Recurrence quantification analysis (RQA) characterizes the dynamics of state change in a recurrence plot. A recurrence plot is composed of recurrence points, each reflecting the returning of the phase space trajectory of the sEMG signal to a previous state, in accordance with the criterion in Equation (7):


$$R_{i,j}=\Theta (\varepsilon -∥{\vec{x}}_{i}-{\vec{x}}_{j}∥)$$ (7)

where ${\vec{x}}_{i}=[u_{i},u_{i+\tau },\ldots u_{i+(m-1)\tau }]$ is the phase space trajectory at the *i*th sampling point, with embedding dimension m and time delay τ ; ‖⋅‖ is the Euclidian distance; and Θ is the Heaviside function that equals 1 when $∥{\vec{x}}_{i}-{\vec{x}}_{j}∥\leq \varepsilon$ and 0 when $∥{\vec{x}}_{i}-{\vec{x}}_{j}∥>\varepsilon$ . A recurrence plot is defined as a state when $∥{\vec{x}}_{i}-{\vec{x}}_{j}∥\leq \varepsilon$ .

Recurrent plots were generated for stationary 1-s segments of each sEMG channel, with parameters set to m=30 , τ=5 , and ε=0.1 , as determined by standard methods (i.e., mutual information and false nearest neighbors). Two complexity descriptors—recurrence rate (RR) and determinism (DET)—were calculated from each recurrence plot and averaged across all plots for each channel. RR is defined as the ratio of recurrence points to all possible states in the plot, while DET is defined as the ratio of recurrence points forming diagonal structures to all recurrence points, reflecting the periodicity of the signal. Higher values of RR and DET reflect greater recurrence and periodicity, and consequently, lower complexity of the sEMG signal.

#### 2.3.5 Graph network analysis

Graph network analysis was applied to the whole muscle network, encompassing six nodes (muscles) interconnected by weighted edges. Adapted from a prior proof-of-concept study (Rong et al., 2025), this analysis selectively extracted a subset of features from the original large feature space based on their demonstrated sensitivity and robustness in detecting distinct neuromuscular changes within the muscle network at both node and edge levels in individuals with ALS. Specifically, at the node level, each sEMG signal was first transformed to a local standard deviation series using Equation (8):


$$V_{j}=\sqrt{\frac{\sum_{(j-1)*L+1}^{\mathrm{jL}}{\left(U_{i}-{\bar{U}}_{j}\right)}^{2}}{L-1}}$$ (8)

where $\left[U_{j}]=[U_{1},U_{2},\ldots ,U_{N}\right]$ is the original sEMG signal, $\left[V_{j}]=[V_{1},V_{2},\ldots ,V_{M}\right]$ is the transformed local standard deviation series, with the interval length set as L=100 (i.e., 50 ms). Next, the local standard deviation series were converted into visibility graphs, where each data point served as a node and the connections between nodes were determined according to the criterion in Equation (9):


$$\frac{V_{y}-V_{z}}{y-z}>\frac{V_{y}-V_{x}}{y-x}$$ (9)

If all intermediate nodes $V_{z}$ between two given nodes $V_{x}$ and $V_{y}$ satisfied this criterion, $V_{x}$ and $V_{y}$ were considered connected. Based on the visibility graphs, density—defined as the ratio of the total number of edges to the largest possible number of edges in the graph—was calculated. This graph descriptor quantifies the visibility of bursts, such that reduced homogeneity of myoelectric activity during syllable repetitions—reflecting a potential DDK deficit in individuals with neuromotor disorders—results in increased burst visibility.

At the edge level, edge weights were computed based on intermuscular coherence within three frequency bands: *θ*/*α* (4–12 Hz), *β* (12–30 Hz), and low-*γ* (30–60 Hz). These frequency bands correspond to distinct neural oscillations for modulating muscle contractions: (i) fast oscillations in the β and low-γ bands originate from the motor cortical network, modulating submaximal tonic contraction and attentionally more-demanding, stronger tonic and phasic contractions, respectively (Boonstra et al., 2015; Brown, 2000); and (ii) slow oscillations in the θ/α band arise from diverse sources outside the motor cortical network (e.g., brainstem), contributing to muscle contractions via indirect mechanisms (MacKay, 1997).

To compute intermuscular coherence, each sEMG signal was full-wave rectified and reconstructed by concatenating stationary 1-s epochs centered on the bursts. The magnitude-squared coherence spectrum was calculated for each pair of reconstructed signals using 4,096-point FFT across 1,024-point, 75% overlap Hamming windows using Equation (10):


$${|R_{\mathrm{xy}}|}^{2}=\frac{{|S_{\mathrm{xy}}(f)|}^{2}}{S_{\mathrm{xx}}(f)S_{\mathrm{yy}}(f)}$$ (10)

where ${|R_{\mathrm{xy}}|}^{2}$ is magnitude-squared coherence spectrum; $S_{\mathrm{xy}}(f)$ is the cross-spectrum between two muscles; and $S_{\mathrm{xx}}(f)$ and $S_{\mathrm{yy}}(f)$ are the auto-spectra for the muscles. A significance level corresponding to the upper 95% confidence limit under the hypothesis of independence between muscles was computed as: $S=1-0.05^{1/(\hat{L}-1)}$ , where $\hat{L}$ is the adjusted number of overlapped segments during the calculation of the coherence spectrum. Using this significance threshold, all spurious, non-significant coherence values within the targeted bands were set to zero. After verifying significance, the Fisher z-transformation of the mean coherence within each band was calculated to serve as the weight of each edge. Based on the weights of all edges, two standard network metrics—mean nodal strength and global efficiency—were derived for each target band. Mean nodal strength, defined as the average sum of edge weights across nodes, reflects the overall functional connectivity of the network. Global efficiency, defined as the average inverse shortest path length between all pairs of nodes, represents the overall functional integration of the network.

### 2.4 Statistical analyses

All statistical analyses were conducted in the R Statistical Computing program (R Core Team, 2024) with a total sample size of *N* = 104 (26 participants × 4 recordings). Prior to analyses, all sEMG features underwent standard data cleaning and imputation procedures. Data points falling outside the range of [lower quantile – 1.5* IQR, upper quantile + 1.5*IQR] were identified and inspected to determine whether they represented outliers or disease-related variability; all outliers were removed, and the corresponding values were imputed using the nonparametric *classification and regression tree (CART)* method (van Buuren and Groothuis-Oudshoorn, 2011).

#### 2.4.1 Effects of ALS on sEMG features

Each sEMG feature was analyzed using a linear mixed-effects model (Kuznetsova et al., 2017), with group (ALS vs. HC) serving as a fixed effect and a subject-dependent intercept included as a random effect. Cohen’s d effect sizes were calculated from the estimated marginal means of the group difference for each feature (Lenth, 2020).

#### 2.4.2 Outcome measures of the sEMG framework

To reduce dimensionality, the 60 sEMG features were clustered into a lower-dimensional set of factors using factor analysis with the *minimal residual* factoring method and *promax* rotation (Revelle, 2020). The number of factors was determined by parallel analysis (Hayton et al., 2004). Features with absolute loadings greater than 0.40 on a given factor were regarded as its component features, reflecting the latent constructs represented by that factor. Factor scores were estimated using the *Ten Berge* method, yielding a set of composite measures indicating individuals’ standing on the latent constructs. These composite measures served as the primary outcome measures for the sEMG framework in the subsequent analyses.

#### 2.4.3 Validation of the sEMG framework

To evaluate the reliability of the sEMG framework, the internal consistency of all composite measures was calculated using Cronbach’s α . To assess the validity of the framework, the associations between all composite measures and three standardized functional metrics—ALSFRS-R bulbar subscore, speech intelligibility, and speaking rate—as well as one biomechanical metric, stiffness, operationally defined as the ratio of jaw range of motion to maximum speed, were evaluate using mediation analysis. The three standardized functional metrics were combined into an integrated outcome measure through the transformations in Equation (11):


$$\begin{array}{l} \text{Out}=c1*z\;(\text{Bulb}\_\text{ALSFRS})+\\ c2*z+(\log_{10}(\max\;(\text{Intell}+1)-\text{Intell}\;))+\\ c3*z(1/\mathrm{SR}) \end{array}$$ (11)

where *Bulb_ALSFRS*, *Intell*, and *SR* are the ALSFRS-R bulbar subscore (with healthy controls assigned the maximum score of 12, corresponding to no bulbar impairment), intelligibility, and speaking rate, respectively; *z* represents the z-score transformation of each metric; and *c1 – c3* are linear combination weights determined by the mediation analysis. The transformations were aimed at enhancing the consistency of data distributions across the three functional metrics.

The mediation analysis examined direct and stiffness-mediated indirect associations between the composite measures and functional outcomes. Stiffness is a key biomechanical property modulated by both central and peripheral nervous systems to enable precise and fast movements of musculoskeletal structures (Moore et al., 1988). It was selected as a mediator of the association between the composite sEMG measures and functional outcomes because prior studies have independently demonstrated its linkages to both neuromuscular properties and functional performance of the bulbar/speech motor system (Rong et al., 2025; Saltzman and Munhall, 1989).

#### 2.4.4 Efficacy of the sEMG framework for detecting prodromal and symptomatic bulbar involvement in ALS

All composite measures were fed into two classification algorithms—mixture discriminant analysis and support vector machine with the radial basis function kernel—to perform multiclass classification of the 104 samples across three groups (i.e., ALS+B, ALS-B, HC), using leave-one-out cross-validation. The overall classification performance was evaluated via mean accuracy, sensitivity, specificity, positive predictive value, and negative predictive value. In addition, pairwise classification performance was assessed using multiclass receiver operating characteristic (ROC) curves and the corresponding areas under the curve (AUC).

To further evaluate the robustness of classification performance against common confounders, all composite measures were adjusted for age, biological sex, and cognitive status (i.e., normal versus mild cognitive impairment, based on a MoCA cutoff score of 26) and subsequently analyzed using the same classification algorithms. Pre- and post-adjustment classification performance was compared.

## 3 Results

### 3.1 Effects of ALS on sEMG features

**Figure 1:**
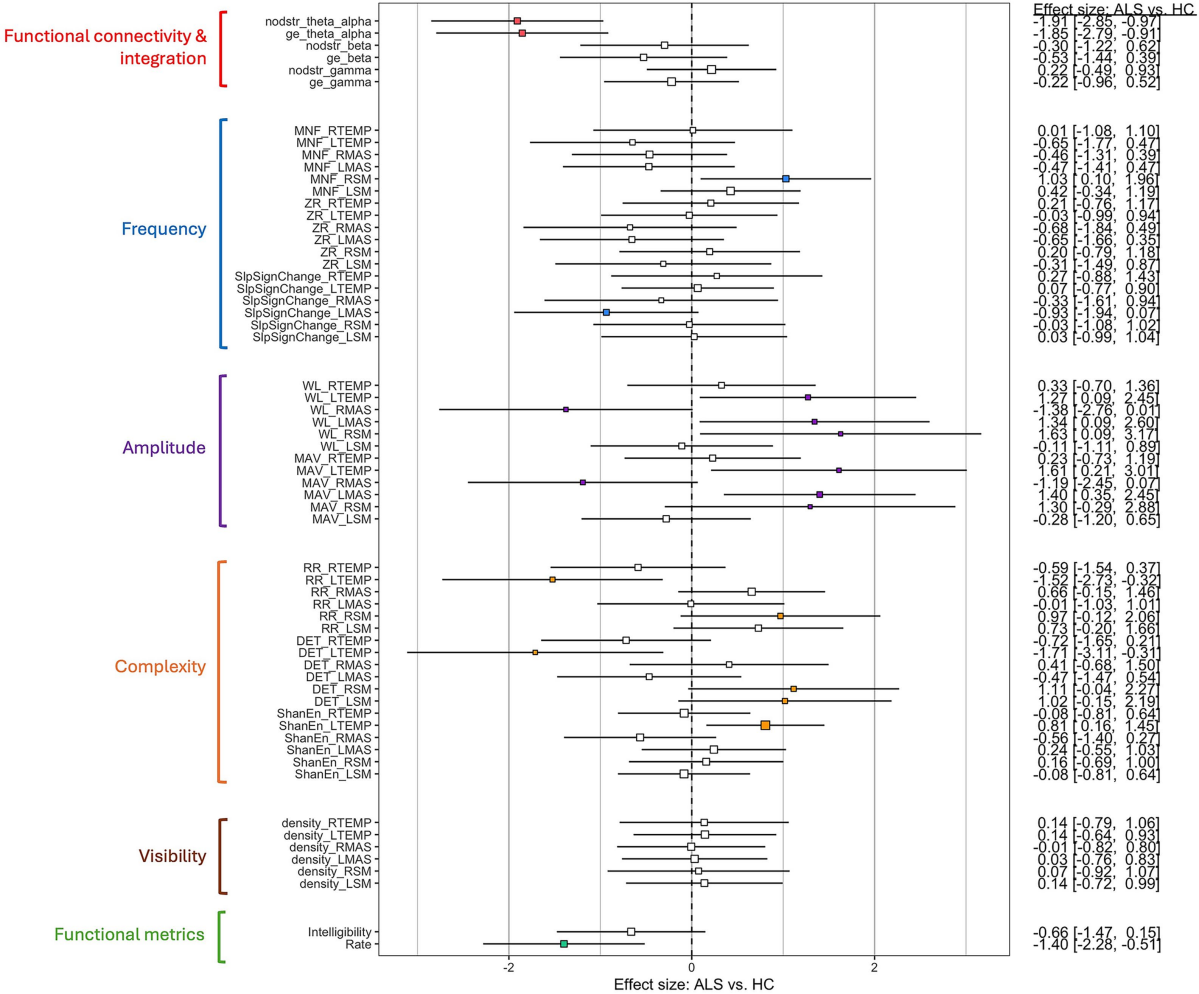
Cohen’s *d* effect sizes for group differences in surface electromyography features and bulbar functional metrics (Intelligibility: speech intelligibility in percentage of intelligible words; Rate: speaking rate in words per minute) between the amyotrophic lateral sclerosis (ALS) and healthy control (HC) groups. Features are grouped by construct, annotated with distinct colors on the left side of the plot. The squares and horizontal bars denote the mean and 95% confidence intervals of the effect sizes, respectively. Large effects (>0.80) are marked by filled squares.

The effect sizes for group differences in the sEMG features and functional metrics between the ALS and HC groups are displayed in Figure 1. The ALS group exhibited both global and localized changes in craniofacial neuromuscular performance. Globally, there was a decrease in functional connectivity and integration, most pronounced in the low frequency band ( θ/α ). Locally, differential disease effects were observed across muscle groups; the primary disease-related changes included: (1) reduced frequency but increased amplitude and complexity of myoelectric activity in the left agonists (temporalis and masseter); (2) reduced amplitude and complexity of the right masseter activity; (3) increased frequency and amplitude but reduced complexity of the right submental complex activity; and (4) reduced complexity of the left submental complex activity.

### 3.2 Factor analysis

The sEMG features were clustered into 10 factors, together accounting for 75.44% of the variance in the original feature space. The rotated factor loadings are provided in Appendices. The component features of each factor, identified based on the loading patterns, are summarized in Table 3, along with their associated latent constructs. All factors were interpretable, reflecting either global or muscle-specific neuromuscular characteristics.

**Table 3:** Clustering of surface electromyography features based on factor analysis.

Factor	Component features	Latent constructs
1: AmpComp_Lag	MAV_LTEMP, MAV_LMAS, WL_LTEMP, WL_LMAS, RR_LTEMP, RR_LMAS, DET_LTEMP, DET_LMAS, ShanEn_LTEMP, ShanEn_LMAS	Amplitude and complexity of left agonist activities
2: Visibility	density_RTEMP, density_LTEMP, density_RMAS, density_LMAS, density_RSM, density_LSM	Visibility of myoelectric bursts
3: Comp_antag	RR_RMAS, RR_RSM, RR_LSM, DET_RMAS, DET_RSM, DET_LSM, ShanEn_RMAS, ShanEn_RSM, ShanEn_LSM	Complexity of antagonist activities
4: Freq_antag	MNF_RMAS, MNF_RSM, MNF_LSM, ZR_RMAS, ZR_RSM, ZR_LSM, SlpSignChange_RMAS, SlpSignChange_RSM, SlpSignChange_LSM	Frequency of antagonist activities
5: Freq_Lag	MNF_LTEMP, MNF_LMAS, ZR_LTEMP, ZR_LMAS, SlpSignChange_LTEMP, SlpSignChange_LMAS	Frequency of left agonist activities
6: AmpComp_RTEMP	MAV_RTEMP, WL_RTEMP, RR_RTEMP, DET_RTEMP, ShanEn_RTEMP	Amplitude and complexity of the right temporalis activity
7: FcInt	nodstr_theta_alpha, nodstr_beta, ge_theta_alpha, ge_beta	Functional connectivity and integration in the θ/α and β bands
8: Amp_RMAS	MAV_RMAS, WL_RMAS	Amplitude of the right masseter activity
9: Freq_RTEMP	MNF_RTEMP, ZR_RTEMP, SlpSignChange_RTEMP	Frequency of the right temporalis activity
10: Amp_SM	MAV_RSM, MAV_LSM, WL_RSM, WL_LSM	Amplitude of submental complex activities

### 3.3 Reliability and validity of the sEMG framework

**Figure 2:**
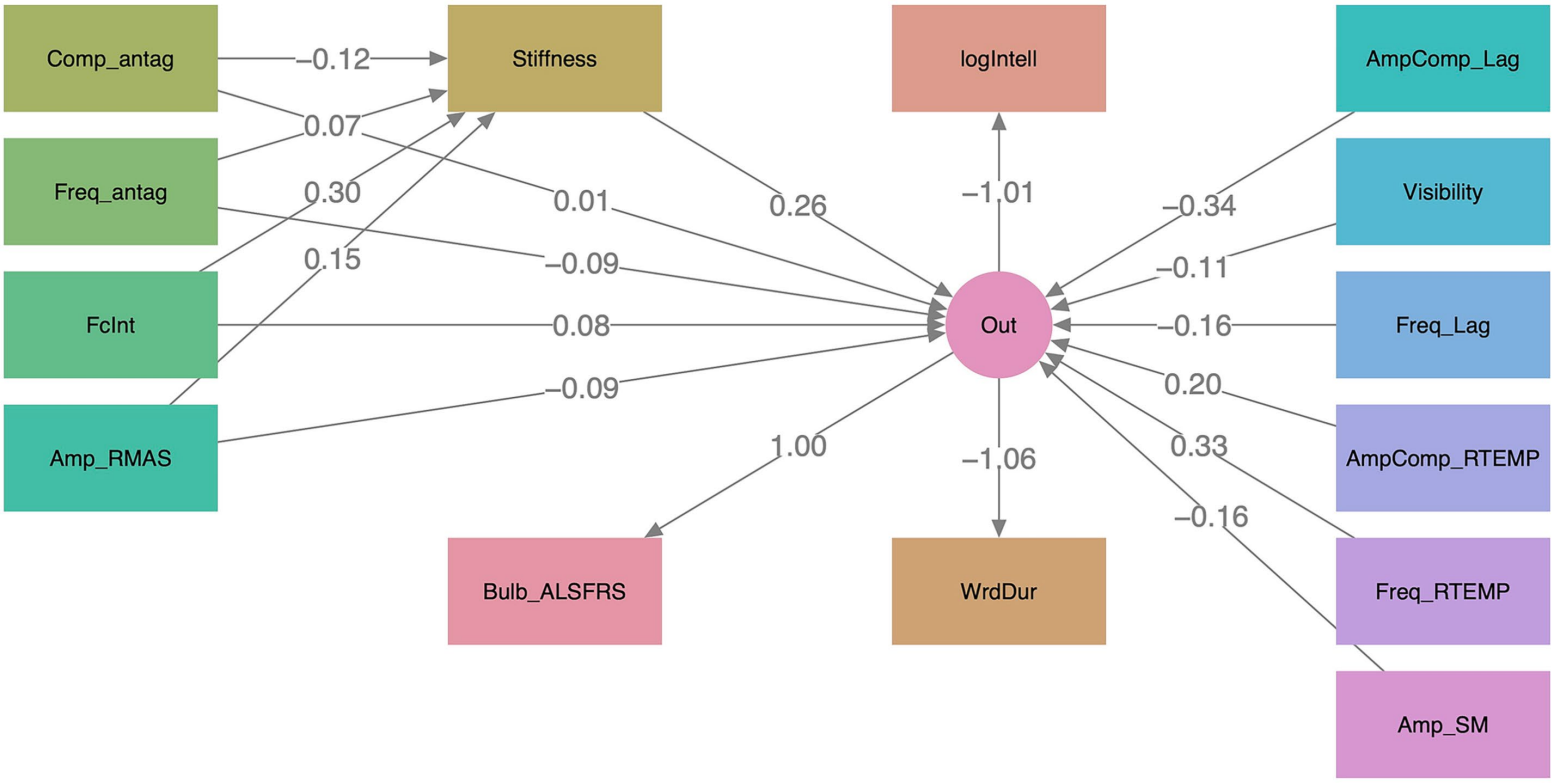
Path diagram for mediation analysis. Among the 10 composite measures derived from the surface electromyography framework, all except one (*Visibility*) exhibited significant direct or indirect relationships with the integrated functional outcome (*Out*). Coefficients for all relationships are displayed along the connecting paths. Indirect relationships were observed through stiffness-mediated pathways involving four composite measures: (1) *Comp_antag* (complexity of antagonist activities), (2) *Freq_antag* (frequency of antagonist activities), (3) *FcInt* (functional connectivity and integration), and (4) *Amp_RMAS* (amplitude of the right masseter activity). Direct relationships were identified for: (1) *AmpComp_Lag* (amplitude and complexity of left agonist activities), (2) *Freq_Lag* (frequency of left agonist activities), (3) *AmpComp_RTEMP* (amplitude and complexity of the right temporalis activity), (4) *Freq_RTEMP* (frequency of the right temporalis activity), and (5) *Amp_SM* (amplitude of submental complex activities). The remaining composite measure, *Visibility* (visibility of myoelectric bursts), exhibited a marginally significant direct relationship with *Out*. The integrated functional outcome comprised three standardized or transformed bulbar functional metrics: Bulb_ALSFRS (bulbar subscore on the Amyotrophic Lateral Sclerosis Functional Rating Scale-Revised), logIntell (log-transformed speech intelligibility), and WrdDur (inversely transformed speaking rate, expressed as mean word duration). The transformations were applied to enhance the consistency of data distributions across the functional metrics.

All composite measures were internally consistent, Cronbach’s α=0.89±0.071 (range: 0.71–0.98). The mediation model exhibited a good fit, CFI = 0.94, RMSEA = 0.098, SRMR = 0.032, with its path diagram displayed in Figure 2. Nine of the 10 composite measures revealed significant direct or indirect associations ( p<0.05 ) with the integrated bulbar functional outcome that combined the ALSFRS-R bulbar subscore, speech intelligibility, and speaking rate. The indirect relationships were mediated by stiffness, wherein reduced frequency and complexity of the antagonist activities, diminished functional connectivity and integration, as well as decreased amplitude of the right masseter activity were associated with reduced stiffness and, consequently, degraded functional outcomes. Increased amplitude, complexity, and frequency of the left agonist activities, reduced frequency but increased amplitude and complexity of the right temporalis activity, as well as increased amplitude of the submental complex activities were directly associated with degraded functional outcomes. Additionally, increased visibility exhibited a marginally significant direct association with degraded functional outcomes ( p=0.054 ).

### 3.4 Efficacy of the sEMG framework for detecting prodromal and symptomatic bulbar involvement in ALS

**Figure 3:**
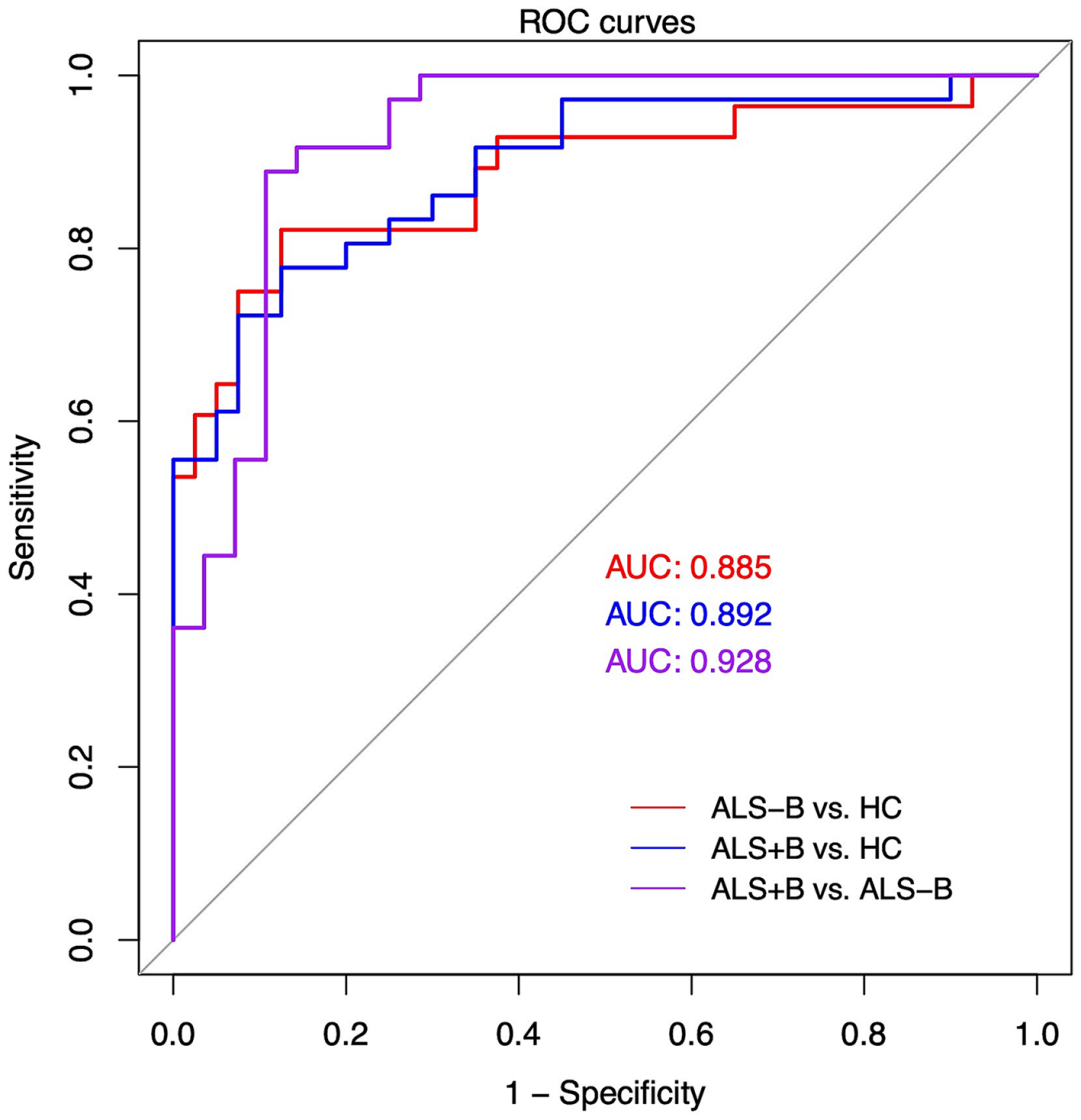
Multiclass receiver operating characteristic (ROC) curves for support vector machine distinguishing ALS-B from HC (red), ALS+B from HC (blue), and ALS+B from ALS-B (purple), based on the composite measures derived from the surface electromyography framework. ALS+B (amyotrophic lateral sclerosis with overt bulbar symptoms); ALS-B (amyotrophic lateral sclerosis without overt bulbar symptoms); HC (healthy control); AUC: area under the curve.

The performance metrics for multiclass classification are provided in Table 4. Both classification algorithms demonstrated consistent performance, which remained stable after adjustment for age, sex, and cognitive status. Figure 3 displays the multiclass ROC curves for pairwise classifications among ALS+B, ALS-B, and HC, generated using the support vector machine algorithm as an illustrative example (the mixture discriminant analysis exhibited similar performance and was thus not shown). All pairwise classifications demonstrated high accuracy, as indicated by the AUC values (>0.90).

**Table 4:** Performance metrics for multiclass classification among ALS+B (amyotrophic lateral sclerosis with overt bulbar symptoms), ALS-B (amyotrophic lateral sclerosis without overt bulbar symptoms), and HC (healthy control) groups, based on the composite measures derived from the surface electromyography framework before and after adjustment for age, biological sex, and cognitive status (adjusted results in parentheses).

Algorithm	Accuracy	Sensitivity	Specificity	PPV	NPV
MDA	0.84 (0.85)	0.84 (0.84)	0.92 (0.92)	0.83 (0.85)	0.92 (0.92)
SVM	0.84 (0.82)	0.84 (0.81)	0.91 (0.90)	0.85 (0.83)	0.92 (0.91)

MDA, mixture discriminant analysis; SVM, support vector machine; PPV, positive predictive value; NPV, negative predictive value.

## 4 Discussion

A craniofacial sEMG framework was developed, which integrates a standardized, repeatable protocol with a fully automated analytic pipeline to enable objective assessment and characterization of bulbar involvement in ALS. This framework demonstrated strong reliability and validity, generating 10 internally consistent objective measures that captured clinically meaningful neuromuscular changes associated with both mechanistic and functional alterations in the bulbar motor system. Moreover, this framework outperformed standard clinical evaluations in detecting subclinical bulbar involvement during the prodromal phase. Collectively, the findings support the potential of the craniofacial sEMG framework as a valuable complement to standard clinical evaluations by providing a clinically applicable, objective, and quantitative tool to improve the early detection of prodromal bulbar involvement and facilitate measurement-based care in ALS.

### 4.1 Effects of ALS on craniofacial neuromuscular performance

The group comparisons in Figure 1 revealed a variety of interpretable changes in craniofacial neuromuscular performance in individuals with ALS. These changes reflect both global impacts of bulbar involvement on the integrated muscle network and localized, muscle-specific effects on segregated network components. Global changes include decreases in functional connectivity and integration, most prominently within the θ/α band and, to a lesser extent, within the β band. One possible source of θ/α oscillations is the brainstem circuitry—including the central pattern generators (CPGs)—which regulate rhythmic, patterned behaviors (MacKay, 1997). Degeneration of brainstem LMNs can disrupt CPG function, resulting in irregular muscle activation patterns, and consequently, decreased functional connectivity and integration of the muscle network within the θ/α band. In contrast, degeneration of UMNs and the corticobulbar tract can disrupt cortically originated β oscillations involved in synchronous modulation of functionally related muscle contractions, leading to reduced functional connectivity and integration within the β band (Rong and Pattee, 2021). Collectively, these neuromuscular changes can be attributed to the combined effects of both UMN and LMN pathologies. These findings are overall consistent with those of Rong et al. (2025), with the exception that the present study did not find a decrease in γ -band functional connectivity and integration. This is likely because of task differences: oral DDK tasks are cognitively less demanding than the oral reading task used by Rong et al. (2025) and are expected to preferentially engage θ/α - and β -band modulations over γ -band modulation, making γ -band functional connectivity and integration less susceptible to disease effects.

At the local level, bulbar involvement led to muscle-specific changes that varied in accordance with physiological characteristics, functional roles, and laterality. Among all three muscle groups, the submental complex—containing the greatest proportion of vulnerable Type II fibers (Korfage et al., 2005)—has been shown in prior studies to be most susceptible to neurodegeneration (Pun et al., 2006). Consistently, we identified the most profound changes in the submental complex. These changes—including increased frequency and amplitude, as well as reduced complexity—imply increased recruitment of larger, fast-conducting motor units, likely as an adaptive response to the progressive loss of viable motor units and reduced firing rates that together diminish the force-generating capacity of the neuromuscular system (de Carvalho et al., 2012). This recruitment pattern, consistent with the size principle (Henneman, 1980), may reflect an attempt to maintain sufficient force as neuromuscular capacity declines.

Among the agonist muscles for jaw elevation (i.e., temporalis and masseter), bulbar involvement exhibited a greater effect on the masseter than the temporalis and preferentially involved the right-sided muscles compared to the left. The observed decreases in the amplitude and complexity of the right masseter activity could be attributed to reduced voluntary recruitment and loss of viable motor neurons associated with both UMN and LMN pathologies. Such changes, however, were not observed in the right temporalis, likely due to its higher proportion of Type I fibers (Korfage et al., 2005), which are the most resistant to neurodegeneration among all fiber types (Pun et al., 2006). The differential disease effects on the right masseter versus the right temporalis align with the findings of Rong and Pattee (2022) obtained from a different task (i.e., oral reading).

Furthermore, the left agonists exhibited distinct changes relative to their right counterparts, which were characterized by increased amplitude and complexity, and reduced frequency. A comparison of the disease-related changes in the left and right agonists imply reduced activation of the right agonists (especially masseter) accompanied by increased engagement of the left agonists. Such a laterality-related effect has also been reported elsewhere (Rong et al., 2025), presumably reflecting the interplay between the preferential left hemispheric involvement in ALS (Henderson et al., 2019) and the contralaterally dominant corticobulbar innervation for craniofacial muscles (Butler et al., 2001). Consequently, the right-sided muscles tend to be more vulnerable than their left-sided counterparts, leading individuals with ALS to increasingly recruit the left-sided muscles to compensate for the right-sided deficits.

### 4.2 Linkages of the sEMG framework to mechanistic and functional alterations

The sEMG framework yielded 10 internally consistent, interpretable composite measures that demonstrated direct or indirect associations with the functional outcomes. The stiffness-mediated pathways further linked disease-related neuromuscular changes to biomechanical alterations in the musculoskeletal system. As shown in Figure 2, reduced CPG- and cortically modulated muscle coordination—indicated by decreased functional connectivity and integration of the muscle network within both θ/α and β bands (*FcInt*)—was associated with reduced stiffness. Oral DDK tasks are conventionally considered “quasi-speech,” positioned between CPG-modulated rhythmic nonspeech tasks (e.g., chewing) and cortically modulated connected speech tasks (Ziegler, 2003). The associations between stiffness and both CPG- and cortically modulated muscle coordination patterns are consistent with this quasi-speech nature, confirming that impairments in both neural sources contribute to reduced stiffness and thereby compromise the musculoskeletal system’s capacity for generating rapid muscle contractions during oral DDK tasks.

In addition, the progressive loss of viable motor units within the submental complex and right masseter—manifested by reductions in the complexity of antagonist activities (*Comp_antag*) and the amplitude of the right masseter activity (*Amp_RMAS*)—was linked to reduced stiffness. Conversely, the recruitment of fast-conducting motor units within the submental complex, as indicated by increased frequency in the antagonist group (*Freq_antag*), was associated with increased stiffness, likely representing an adaptive mechanism to partially offset the disease-related decrease in stiffness. These findings align with the critical functional role of antagonists in stiffening musculoskeletal structures to support the generation of rapid, precise movements (Moore et al., 1988). Because slow and imprecise articulatory movements are major contributors to functional speech decline in ALS (Moore and Rong, 2022; Rong et al., 2016), the indirect associations identified here elucidate mechanistic pathways that link neuromuscular impairments and adaptations with biomechanical changes (stiffness) and subsequent alterations in functional outcomes (speaking rate and speech intelligibility) in ALS.

The direct associations observed between neuromuscular and functional changes are likely attributed to two factors: the severity of bulbar involvement and neuromuscular fatigue. The associations between the amplitude- and complexity-based features and functional outcomes may reflect severity-modulated neuromuscular adaptations and their downstream effects on functional performance. Among the agonist muscles, these adaptations include increased activation of the left-sided agonists relative to their right-sided counterparts and greater engagement of the temporalis relative to the masseter, as indicated by increased amplitude and/or complexity in the left agonists (*AmpComp_Lag*) and right temporalis (*AmpComp_RTEMP*). Within the submental complex, adaption appears to be achieved through increased recruitment of larger motor units, as reflected by increased amplitude (*Amp_SM*). Collectively, these changes in *AmpComp_Lag*, *AmpComp_RTEMP*, and *Amp_SM*, in conjunction with concurrent declines in functional outcomes, are consistent with severity-modulated effects on both neuromuscular and functional performance.

The observed associations between frequency-based features and functional outcomes may reflect the interplay between bulbar severity and neuromuscular fatigue. Specifically, increased engagement of the left-sided agonists and the right temporalis can increase muscle fatiguability, leading to reduced frequency—a common sign of neuromuscular fatigue (Yaar and Niles, 1992)—and consequent declines in functional outcomes. In addition to this fatigue-modulated effect, the frequency of the masseter activity—which is typically more vulnerable than the temporalis due to its higher proportion of Type II fibers—can be further modulated by bulbar severity. As severity increases, continued loss of viable motor units in the masseter necessitates a shift in recruitment toward larger, fast-conducting motor unit pools following the size principle to meet force-generation demands, resulting in increased frequency accompanied by declining functional outcomes. Taken together, the association between *Freq_RTEMP* and functional outcomes may reflect predominantly fatigue-modulated effects, whereas the association between *Freq_Lag* and functional outcomes is more likely to capture the combined effects of bulbar severity and neuromuscular fatigue on the temporalis and masseter.

Finally, the marginally significant association between increased visibility and degraded functional outcomes may also reflect a severity-modulated effect. Different from connected speech, in which greater visibility of myoelectric bursts—reflecting the dynamics of speech production—is a desirable characteristic reflecting natural prosodic variations (e.g., grammatical and emotional stress), oral DDK tasks do not contain such higher-level linguistic and paralinguistic content. Therefore, increased visibility of myoelectric bursts during oral DDK tasks is more likely to reflect a distinct phenomenon, indicative of impaired neuromuscular control in generating regular, repetitive muscle activation patterns for syllable repetition, as commonly observed in ALS (Rong, 2020; Rong et al., 2018). Such irregularity of muscle activation patterns can arise from (1) the recruitment of increasingly heterogeneous motor unit pools, including units reinnervated through collateral sprouting, (2) increased variability of motor unit firing behaviors (de Carvalho et al., 2014), and (3) greater complexity and instability of motor unit action potential morphology originating from chronic denervation and reinnervation (Costa et al., 2012). As the severity of bulbar involvement increases, the irregularity of muscle activation patterns also increases (Rong et al., 2018). The associated declines in functional outcomes can therefore be interpreted as downstream effects of this neuromuscular deficit on functional performance.

### 4.3 Added value of the sEMG framework over standard clinical evaluations

Both machine learning algorithms demonstrated high performance in distinguishing clinically confirmed (ALS+B) and silent (ALS-B) cases from healthy controls. These results suggest that the sEMG framework is sensitive to bulbar neuromuscular changes across both prodromal and symptomatic phases, enabling earlier detection of bulbar involvement compared with standard clinical evaluations. In addition, the sEMG framework showed strong discriminatory efficacy in distinguishing ALS+B from ALS-B (Figure 3), indicating its responsiveness to dynamic changes during the transition from prodromal to symptomatic phases. With further longitudinal validation warranted, this finding underscores the potential of the sEMG framework for monitoring the progression of bulbar involvement.

Notably, the performance of the classification models remained stable after adjustment for age, sex, and cognitive status. Age- and sex-related variability in anatomical, physiological, and biological characteristics can introduce disease-unrelated variations to neuromuscular performance. Additionally, changes in higher-order language and cognitive processes due to frontotemporal lobar degeneration may further confound the interpretation of speech-based measures, as shown in prior work (Yunusova et al., 2019). To minimize the effects of these common disease-related and unrelated confounders, we carefully designed the sEMG framework by selecting “quasi-speech” tasks that preserve the strengths of speech, while reducing cognitive-linguistic demands. Our findings provide corroborative evidence for the robustness of this framework to the aforementioned confounders that challenge speech-based bulbar measurements. Taken together, the pre- and post-adjustment classification results support the potential of the sEMG framework as a robust and efficacious objective tool to complement standard clinical evaluations by detecting subtle yet meaningful subclinical bulbar motor changes while remaining resistant to nonmotor confounders.

### 4.4 Clinical implications

The proposed sEMG framework has several clinical implications. Given its sensitivity to subclinical bulbar neuromuscular changes, the framework can improve early identification and referral of asymptomatic individuals during the prodromal phase of bulbar involvement. This will afford patients and their caregivers valuable time to gather information, consider management options (e.g., voice banking), and explore therapeutic trial opportunities, thereby supporting informed decision-making and patient-centered care before the presentation of pronounced bulbar symptoms. Furthermore, the sEMG framework offers objective and interpretable outcome measures that may function as surrogate biomarkers for evaluating therapeutic efficacy in clinical trials. Additionally, these markers can inform measurement-based behavioral interventions, enabling dynamic tailoring of interventions to patients’ evolving needs throughout the disease course.

### 4.5 Limitations and future directions

Several limitations of this study must be acknowledged. Given the relatively small sample size and the inherent heterogeneity of ALS, the results should be considered exploratory and might not be directly generalizable to the broader patient population. To mitigate these limitations, we carefully selected data-driven approaches: support vector machine is a robust machine learning algorithm well suited for small datasets, while mixture discriminant analysis is designed to accommodate intrinsic within-class heterogeneity by modeling each class as a mixture of Gaussian distributions (Hastie and Tibshirani, 2018). Future studies should focus on the external validation of these classification models with larger, more heterogeneous datasets. In addition, the utility of the sEMG framework for longitudinal monitoring of bulbar progression warrants further investigation.

Bulbar involvement is known to differentially affect muscles with distinct anatomical origins and fiber-type compositions (DePaul et al., 1988). This study employed a selective set of craniofacial muscles with well-established anatomical landmarks to ensure the validity and reproducibility of data collection. Nonetheless, disease effects on other muscles may differ. Expanding the sEMG framework to a broader set of craniofacial muscles would allow us to examine differential disease effects on the bulbar musculature, which should be pursued in future studies.

## 5 Conclusion

The lack of a validated objective assessment tool has been a major barrier to the early diagnosis and effective management of bulbar involvement in ALS. To address this gap, sEMG—a clinically accessible and noninvasive instrument—holds substantial potential. An emerging body of research efforts have been directed toward developing novel analytic approaches to transform the clinical utility of sEMG, which is currently used primarily in an observational and qualitative manner, by extracting clinically relevant quantitative information from craniofacial sEMG signals during controlled speech tasks. However, due to the absence of standardized guidelines, previously investigated tasks have varied widely in their motoric, cognitive, and linguistic demands, limiting direct comparability across studies and hindering translation of the findings to clinical practice. Importantly, the effects of cognitive-linguistic impairments on speech production, evidenced in many speech-based measures, raise questions regarding the validity of these measures as indicators of the underlying neuromotor pathology. The present study is, to our knowledge, the first in the contemporary sEMG literature to address this issue by (1) developing and applying an integrated analytic pipeline to craniofacial sEMG signals acquired during an alternative task paradigm that employs motorically demanding yet cognitively and linguistically less challenging oral DDK tasks, (2) extracting objective, clinically relevant measures of bulbar involvement, and (3) providing empirical evidence demonstrating the robustness of these measures to cognitive-linguistic deficits, along with two other common nonmotor confounders (i.e., age, sex).

Given the simplicity of oral DDK task procedures, the repeatable experimental setup, and the fully automated analytic pipeline, the proposed sEMG framework is highly scalable for clinical implementation, offering an efficient, objective, and quantitative tool for bulbar assessment. Validated in a small ALS cohort with varying severities of bulbar involvement, this tool demonstrated strong reliability and validity, capturing neuromuscular changes linked to meaningful mechanistic and functional alterations in the bulbar motor system. Moreover, this tool outperformed standard clinical evaluations by detecting subclinical neuromuscular changes across both prodromal and symptomatic phases of bulbar involvement. These findings collectively provide compelling preliminary evidence for the utilization of the sEMG framework as a complement to standard clinical evaluations, with the aim of maximizing early assessment and detection and improving measurement-based care of bulbar involvement in ALS.

## Data Availability

The raw data supporting the conclusions of this article will be made available by the authors upon reasonable request and appropriate data sharing agreement.
