# Supplementary material for: Translation of surface electromyography into a clinically applicable objective bulbar assessment tool to improve measurement-based care in amyotrophic laterals sclerosis
Source: Front Neurosci. 2026 Apr 10;20:1784520. doi: 10.3389/fnins.2026.1784520 (PMC13106279; doi:10.3389/fnins.2026.1784520)
Supplement: Supplementary file 1 [file Data_Sheet_1.docx]

Supplementary Material


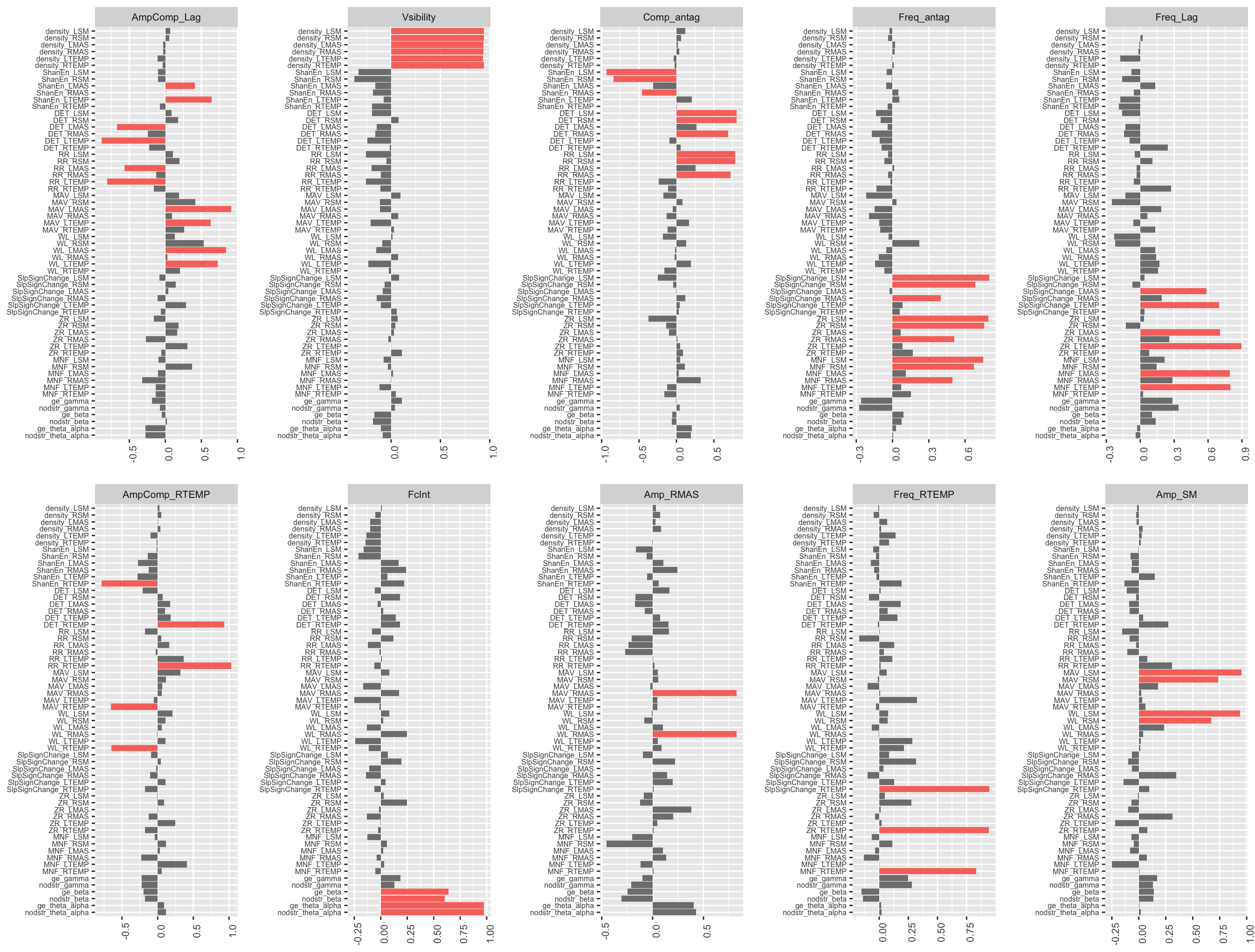


**Supplementary Figure 1.** Rotated factor loadings, indicated by the heights and signs of the bars. Features with absolute loadings greater than 0.40 are highlighted in red; these features comprise the component features of each factor. Note that one feature (i.e., SlpSignChange_LMAS) was initially cross-loaded on two factors—Freq_Lag and Amp_RMAS—with similar loadings (0.59 on Freq_Lag and 0.64 on Amp_RMAS). Although the loading was slightly higher on Amp_RMAS, this feature was assigned to Freq_Lag based on theoretical considerations. This assignment was implemented by adjusting the loadings matrix, and the loadings reported in this future reflect the post-adjustment values. Factor scores were subsequently computed using the adjusted loadings matrix.
